# Supplementary material for: Nursing students and nurses’ knowledge and attitudes regarding children’s pain: A comparative cross-sectional study
Source: PLoS One. 2019 Oct 10;14(10):e0223730. doi: 10.1371/journal.pone.0223730 (PMC6786626; doi:10.1371/journal.pone.0223730)
Supplement: Attachment — Submitted filename: Reviewers Comments_JK.docx [file pone.0223730.s002.docx]

**Title: Nursing Students and Nurses’ Knowledge and Attitudes regarding Children’s Pain: A Comparative Cross-sectional study**

**Reviewer’s comments**

Thank you for the opportunity to review the manuscript.

The manuscript presents original work which is presented in correct language, the methodology and results are in congruence. This piece of work will add to the knowledge in regards to management of pain in children in Ghana.

Tables 1-4 discussions are quite lengthy. There is need to refine the results’ discussion of tables 1-4 to reduce wording, so as to make it easy for the reader.

Regards,

Jane
